# Supplementary material for: Health Information Technology Usability Evaluation Scale (Health-ITUES) and User-Experience Questionnaire (UEQ) for 3D Intraoperative Cognitive Navigation (ICON3DTM) System for Urological Procedures
Source: Medicina (Kaunas). 2023 Mar 21;59(3):624. doi: 10.3390/medicina59030624 (PMC10057936; doi:10.3390/medicina59030624)
Supplement: Supplementary file 1 [file medicina-59-00624-s001.zip › medicina-2233097-supplementary.pdf]

10TH TECHNO-UROLOGY MEETING (TUM) – 28-29 APRIL 2022

ICON3D EXPERIENCE EVALUATION

Only those who complete the entire questionnaire will be listed as part of the collaborative authorship in the future publication (According to ICMJE Recommendation for Authorship).

1. Country: ______________________________________
2. Gender: ☐ Male ☐ Female
3. Position: ☐ Resident ☐ Young Urologist ☐ Senior Urologist (>40 yrs old)
4. Surgical skill (MCQ):

Open ☐ Laparoscopic ☐ Robotic ☐

Health Information Technology Usability Evaluation Scale (5 strongly agree – 1 strongly disagree)

Impact:

5. I think ICON3D has been a positive addition for the patient. 1_2_3_4_5
6. I think ICON3D has been a positive addition for the surgeon. 1_2_3_4_5
7. ICON3D covers an important role in my presurgical/surgical planning and decision making process. 1_2_3_4_5

Perceived usefulness

8. The use of ICON3D makes it easier to understand the patient's and disease's anatomy. 1_2_3_4_5
9. The use of ICON3D enables me to plan my surgical strategy more quickly. 1_2_3_4_5
10. The use of ICON3D makes it more likely for me to rely on standard bidimensional imaging (CT/MRI). 1_2_3_4_5
11. ICON3D is useful for surgical planning or navigation. 1_2_3_4_5
12. I think ICON3D presents a more equitable process for surgical strategy. 1_2_3_4_5
13. I am satisfied with the use of ICON3D for surgical strategy decision-making process (both planning and navigation). 1_2_3_4_5
14. I can consult and study the clinical case in a timely manner because of ICON3D. 1_2_3_4_5
15. Using ICON3D increases the quality (in terms of precision and safety) of my surgical planning and subsequent intervention. 1_2_3_4_5
16. I am able to self-manage surgical planning and navigation whenever I use ICON3D. 1_2_3_4_5

Perceived ease of use

17. I am comfortable with my ability to use ICON3D. 1_2_3_4_5
18. Learning to operate with ICON3D is easy for me. 1_2_3_4_5
19. It is easy for me to become skillful at using ICON3D. 1_2_3_4_5
20. I find ICON3D easy to use. 1_2_3_4_5

21. I can always remember how to log on to and use ICON3D. 1_2_3_4_5

User control

22. ICON3D gives error messages that clearly tell me how to fix problems. 1_2_3_4_5
23. Whenever I make a mistake using ICON3D, I recover easily and quickly. 1_2_3_4_5
24. The information (such as on-line help, on-screen messages, and other documentation) provided with ICON3D is clear. 1_2_3_4_5

<u>Specific ICON3D evaluation section</u>

25. The hands/mouse movements allow to manipulate the 3D model in an intuitive way:
    a. Absolutely yes
    b. Yes, but it takes some times
    c. The software use is complicated and makes me uncomfortable
    d. The software use is impossible and/or the instructions are hard to understand
    e. Absolutely no, it may also be dangerous fot the patient
26. The hands/mouse movements allow the first surgeon to be fully autonomous and independent:
    a. Absolutely yes
    b. It takes some time to become fully autonomous
    c. The software use is complicated and makes me uncomfortable
    d. The software use is impossible and/or the instructions are hard to understand
    e. Absolutely no, it may also be dangerous for the patient
27. How would you rate the under-red interface’s usability (hands movements)? 1_2_3_4_5
28. What was the hardest action/moment during the procedure?
    a. Zooming/tilting the model
    b. Anatomical comprehension
    c. Navigating in the menu and break down the model to its part
    d. Moving/tilting the 3D model synchronously in accordance with the surgical procedure
29. What was the hardest action to execute with the 3D mouse?
    a. Zooming/tilting the model
    b. Anatomical comprehension
    c. Navigating in the menu and break down the model to its part
    d. Moving/tilting the 3D model synchronously in accordance with the surgical procedure
30. Thanks to the software’s windows (user interface) it is possible to find all the necessary information:
    a. Absolutely yes
    b. Yes, but it could be improved
    c. The software use is complicated and makes me uncomfortable
    d. The software use is impossible and/or the instructions are hard to understand
    e. Absolutely no, it may also be dangerous for the patient
31. Does the colors’ choice allow to clearly distinguish the different parts of the model?
    a. Absolutely yes

    b. Yes, but it could be improved
    c. The software use is complicated and makes me uncomfortable
    d. The software use is impossible and/or the instructions are hard to understand
    e. Absolutely no, it may also be dangerous for the patient
32. The input devices would allow easier movements in the operatory field:
    a. Absolutely yes
    b. Yes, but it could be improved
    c. The software use is complicated and makes me uncomfortable
    d. The software use is impossible and/or the instructions are hard to understand
    e. Absolutely no, it may also be dangerous for the patient
33. The use of SW is easy and intuitive:
    a. Absolutely yes
    b. Yes, but it could be improved
    c. The software use is complicated and makes me uncomfortable
    d. The software use is impossible and/or the instructions are hard to understand
    e. Absolutely no, it may also be dangerous for the patient
34. The SW allows to recognize with a high level of accuracy the anatomical elements (e.g., organs, blood vessels) during the different phases of the surgical procedure:
    a. Absolutely yes
    b. Yes, but it could be improved
    c. The software use is complicated and makes me uncomfortable
    d. The software use is impossible and/or the instructions are hard to understand
    e. Absolutely no, it may also be dangerous for the patient
35. It is likely for the SW to become essential during complex surgical procedures:
    a. Absolutely yes
    b. Yes, but it could be improved
    c. The software use is complicated and makes me uncomfortable
    d. The software use is impossible and/or the instructions are hard to understand
    e. Absolutely no, it may also be dangerous for the patient
36. Using the SW, it's easy to make mistakes:
    a. Absolutely yes
    b. Yes, but it could be improved
    c. The software use is complicated and makes me uncomfortable
    d. The software use is impossible and/or the instructions are hard to understand
    e. Absolutely no, it may also be dangerous for the patient
37. Using the SW, it's hard to mistake the anatomy:
    a. Absolutely yes
    b. Yes, but it could be improved
    c. The software use is complicated and makes me uncomfortable
    d. The software use is impossible and/or the instructions are hard to understand
    e. Absolutely no, it may also be dangerous for the patient
38. I consider my ability to use the SW to be satisfactory:
    a. Absolutely yes
    b. Yes, but it could be improved
    c. The software use is complicated and makes me uncomfortable
    d. The software use is impossible and/or the instructions are hard to understand
    e. Absolutely no, it may also be dangerous for the patient
39. The SW use is not time consuming and does not tire out:

a. Absolutely yes
b. Yes, but it could be improved
c. The software use is complicated and makes me uncomfortable
d. The software use is impossible and/or the instructions are hard to understand
e. Absolutely no, it may also be dangerous for the patient

40. Using the SW during the surgical procedure does not stress me out:
    a. Absolutely yes
    b. Yes, but it could be improved
    c. The software use is complicated and makes me uncomfortable
    d. The software use is impossible and/or the instructions are hard to understand
    e. Absolutely no, it may also be dangerous for the patient
41. It’s very easy to locate the lesion’s position and to identify its relations with the surrounding structures:
    a. Absolutely yes
    b. Yes, but it could be improved
    c. The software use is complicated and makes me uncomfortable
    d. The software use is impossible and/or the instructions are hard to understand
    e. Absolutely no, it may also be dangerous fot the patient

<u>PERSONAL DETAILS</u>

1. NAME:
2. SURNAME:
3. INSTITUTION:
4. EMAIL:
